# Palliative Care Service Utilization and Advance Care Planning for Adult Glioblastoma Patients: A Systematic Review

**DOI:** 10.3390/cancers13122867

**Published:** 2021-06-08

**Authors:** Adela Wu, Gabriela Ruiz Colón, Rebecca Aslakson, Erqi Pollom, Chirag B. Patel

**Affiliations:** 1 Department of Neurosurgery, Stanford University School of Medicine, Stanford, CA 94305, USA; 2 Stanford University School of Medicine, Stanford, CA 94305, USA; grc@stanford.edu; 3 Division of Primary Care and Population Health, Department of Medicine, Stanford University School of Medicine, Stanford, CA 94305, USA; aslakson@stanford.edu; 4 Department of Anesthesiology, Perioperative, and Pain Medicine, Stanford University School of Medicine, Stanford, CA 94305, USA; 5 Division of Radiation Therapy, Department of Radiation Oncology, Stanford University School of Medicine, Stanford, CA 94305, USA; erqiliu@stanford.edu; 6 Division of Neuro-Oncology, Department of Neurology & Neurological Sciences, Stanford University School of Medicine, Stanford, CA 94305, USA; 7 Molecular Imaging Program at Stanford (MIPS), Department of Radiology, Stanford University School of Medicine, Stanford, CA 94305, USA

**Keywords:** advance care planning, glioblastoma, caregiver, palliative care, end of life

## Abstract

**Simple Summary:**

Glioblastoma is the most common primary brain malignancy diagnosed in adults, and, despite standard of care treatment, it carries a devastating prognosis with median overall survival of 16–21 months. Advance care planning and palliative care services utilization are important for this patient population due to their cancer and neurocognitive symptoms. We present a systematic review on prevalence of advance care planning, end-of-life services utilization, and experiences among adults with glioblastoma. The findings from our review serve as a foundation for future additional works, particularly prospective studies, that may address gaps in palliative care resource utilization and disparities in advance care planning for adult glioblastoma patients.

**Abstract:**

Glioblastoma (GBM) has a median overall survival of 16–21 months. As patients with GBM suffer concurrently from terminal cancer and a disease with progressive neurocognitive decline, advance care planning (ACP) and palliative care (PC) are critical. We conducted a systematic review exploring published literature on the prevalence of ACP, end-of-life (EOL) services utilization (including PC services), and experiences among adults with GBM. We searched from database inception until 20 December 2020. Preferred reporting items for systematic reviews guidelines were followed. Included studies were assessed for quality using the Newcastle-Ottawa Scale. The 16 articles were all nonrandomized studies conducted in six countries with all but two published in 2014 or later. ACP documentation varied from 4–55%, PC referral was pursued in 39–40% of cases, and hospice referrals were made for 66–76% of patients. Hospitalizations frequently occurred at the EOL with 20–56% of patients spending over 25% of their overall survival time hospitalized. Many GBM patients do not pursue ACP or have access to PC. There is a dearth of focused and high-quality studies on ACP, PC, and hospice use among adults with GBM. Prospective studies that address these and additional aspects related to EOL care, such as healthcare costs and inpatient supportive care needs, are needed.

## 1. Introduction

Glioblastoma (GBM) is the most common primary brain tumor in adults, conferring a grim median overall survival of 16–21 months and a 10-year survival rate of 0.71% [1,2,3,4,5]. Patients with GBM frequently experience challenging physical symptoms and neurologic deficits [6], and suffer from early cognitive decline with consequent impaired executive function [7]. Despite the incurable nature of GBM with an inevitable need for end-of-life (EOL) planning, recent reviews of the glioma and GBM literature have concluded EOL research within this population is underrepresented and requires expansion [8,9].

A recent international panel defined advance care planning (ACP) as a process that “enables individuals to define goals and preferences for future medical treatment and care, to discuss these goals and preferences with family and health-care providers, and to record and review these preferences if appropriate” [10]. ACP is essential for goal-concordant care [11], and is particularly pertinent for GBM patients as they have a terminal cancer and suffer from neurocognitive decline. Among adults with GBM pursuing standard radiation treatment, only 29% had ACP documented within 6 months of diagnosis and approximately half (55%) had ACP documentation before death [12]. In addition, Sborov et al. found that oncologists can be overly-optimistic with inaccurate survival predictions, resulting in hastier EOL care for patients with advanced cancer [13].

Palliative care (PC) is interdisciplinary care focused on improving quality of life for persons with serious illness and is appropriate at any stage of illness [14]. A recent meta-analysis of studies from diverse populations with life-limiting illnesses—70% of which were from cancer populations—supports that proactive PC is associated with improved quality of life and reduced physical and psychological symptom burden [15]. Consequently, guidelines from the American Society of Clinical Oncology (ASCO) recommend that patients with advanced cancer should receive dedicated PC early in their disease course [16].

This review summarizes published data on the prevalence of ACP, healthcare services utilization at the EOL (including PC services), and location of death among adults with GBM. We additionally evaluated for any potential associations between ACP and PC with EOL administrative outcomes and reported outcomes.

## 2. Methods

**Figure 1:**
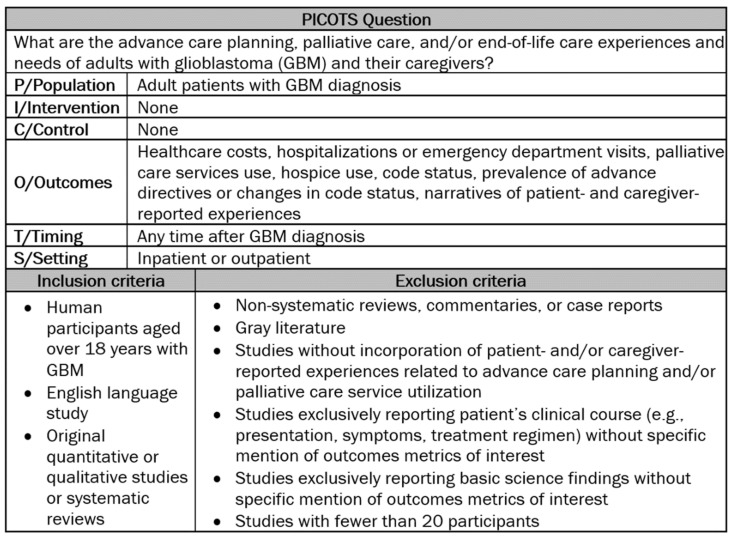
Population, intervention, comparison, outcomes, timing, and study design (PICOTS) question, inclusion criteria, and exclusion criteria.

The literature search was developed by defining the population, intervention, comparison, outcomes, timing, and study design (PICOTS) question, and inclusion and exclusion criteria (Figure 1). The criteria, outcome measures, and search strategy were defined prior to analysis.

### 2.1. Search Strategy

The protocol was designed in accordance with preferred reporting items for systematic reviews and meta-analyses (PRISMA) guidelines [17]. The review included quantitative and qualitative studies of adults with GBM and their caregivers, with at least 20 subjects, and which were written in English. Exclusion criteria included: gray literature, non-systematic reviews, commentaries, and case reports [18]. In collaboration with a university librarian, we deployed a comprehensive search strategy within PubMed, Scopus, Cochrane Library, and Embase databases from inception until 20 December 2020. Synonymous words for key search terms (‘glioblastoma’, ‘end of life’, ‘advance care planning’, ‘advance directive’) were included to maintain high inclusivity of the initial search (Appendix A).

### 2.2. Study Selection

Duplicates were removed and study eligibility was determined using the PICOTS question and inclusion and exclusion criteria (Figure 1). The titles and abstracts, followed by article full text, were independently screened by three study authors (A.W., G.R.C., and C.B.P.) with group discussion used to adjudicate discrepancies.

### 2.3. Data Extraction and Analysis

We extracted key features from the eligible studies, including aims, design, inclusion and exclusion criteria, patient population, and outcomes. Outcome variables included ASCO Quality Oncology Practice Initiative (QOPI) adherence measures, hospice utilization rates, PC utilization rates, percentages of advance directive documentation, and location of death [19].

### 2.4. Quality Assessment

Three authors (A.W., G.R.C., and C.B.P.) independently used the Newcastle-Ottawa Scale to assess the quality of the studies [20]. Studies were graded as very low, low, medium, or high quality based on selection criteria, risk of bias, and overall study design [21]. No studies were excluded from analysis due to quality.

## 3. Results

**Figure 2:**
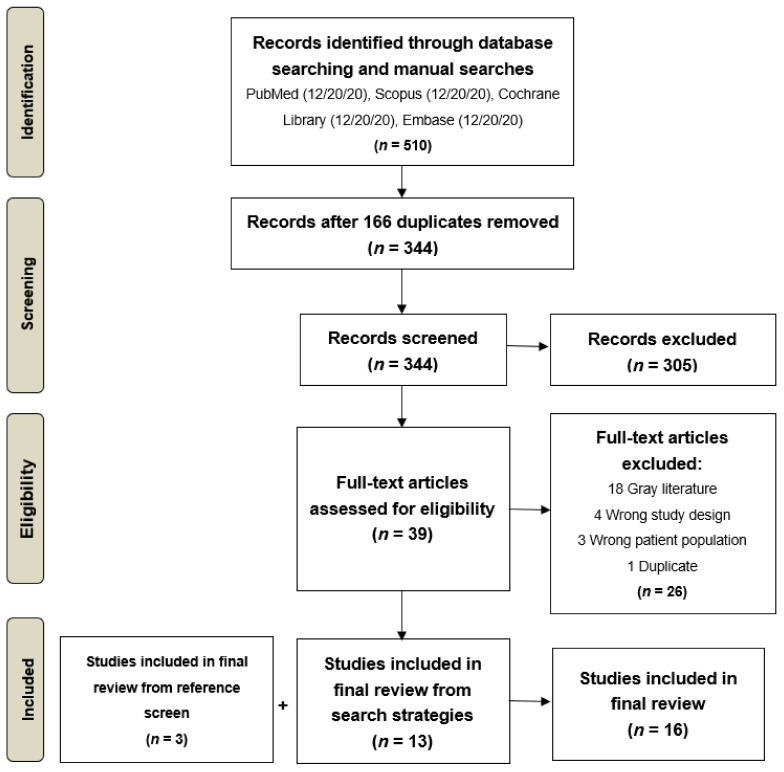
Flow diagram of the systematic review process.

The search yielded 344 unique studies for screening (Figure 2). We excluded 305 publications based on titles or abstracts, and the remaining 39 studies were assessed by full text and included or excluded based on inclusion/exclusion criteria (Figure 1). Of the 39 studies assessed, 18 were excluded because they were gray literature, four had an ineligible study design, three had an ineligible patient population, and one was a duplicate, resulting in 13 studies for inclusion [12,22,23,24,25,26,27,28,29,30,31,32,33]. We also included three additional studies [34,35,36] based on a final review of the references of included articles, resulting in 16 total studies in the final analysis with 10,706 total GBM patients and 123 caregivers. These studies spanned six countries (*N* = 7 USA [12,23,29,30,31,35,36]; *N* = 3 Canada [22,27,34]; *N* = 3 Austria [24,28,33]; *N* = 1 Germany [25]; *N* = 1 Italy [32] and *N* = 1 Australia [26]).

### 3.1. Study Characteristics

Studies were published between 2001 and 2019 with all but two studies published in 2014 or later (Table 1). Ten of the studies were retrospective cohort studies [12,22,23,24,26,29,30,31,34,35], three were cross-sectional studies [27,28,32], two were prospective cohort studies [25,33], and one was a case-control study [36]. Fourteen studies focused on the experiences of GBM patients at the EOL [12,22,23,24,26,27,29,30,31,32,33,34,35,36], and two studies explored outcomes related to caregivers’ experiences [25,28]. The most commonly reported findings were prevalence and/or documentation of ACP conversations (*N* = 7) [12,27,29,30,31,32,33]. Location of death was described in six studies [22,28,29,30,33,37], hospice utilization was described in six studies [12,23,24,29,30,36], and PC utilization in four studies [12,25,26,29]. Both studies which addressed caregiver experience focused on caregiver quality of life, challenges faced, and emotions at the end of the care recipient’s life [25,28].

### 3.2. ACP Conversations and Documentation

The prevalence of any ACP documentation ranged from 4% to 55% (Table 1) [12,29,30,33,34]. The study from our institution by Pollom et al. [12] assessed ACP at different time points in the patients’ treatment course and found that 11% of patients had ACP documented before their diagnosis, 29% within six months after diagnosis, 54% at their last follow-up, and among decedents, 55% prior to death. This prevalence was similar to the rates reported by Hemminger et al. [29], where ACP documentation was available in 52% of cases by the third oncology visit, which is the standard recommended by ASCO QOPI’s EOL performance measures. Three studies also specified the prevalence of code status documentation, ranging from 36% (via a medical orders for life-sustaining treatment {MOLST} form) to 46% [12,29,30]. No study reported the prevalence of ‘do not hospitalize’ directives.

Two studies found the prevalence of an ACP or goals of care conversations to be 83% [27,31]. Miranda et al. [31] found that these conversations most frequently discussed EOL planning (including hospice and PC), prognosis, and prognostic understanding. None of the analyzed conversations reported healthcare proxies, family involvement, or information preferences [31].

### 3.3. Healthcare Services Utilization at the EOL

Ten studies reported healthcare services utilization at the EOL (Table 1) [12,23,24,25,26,29,30,34,35,36]. Four studies addressed PC services referral and utilization [12,25,26,29], six studies addressed hospice referral and utilization [12,23,24,29,30,34] and four studies addressed other healthcare services utilization, including inpatient hospitalizations [30,34,35,36]. Among the four studies reporting PC utilization, PC referrals ranged between 39% and 40% [12,26], and PC consult utilization was reported to be between 34% and 36% [25,29]. Median time from diagnosis to PC consult was only measured in one study and found to be 111 days [26]. Notably, the study by Pollom at al. [12] found that among patients with a PC referral, all had this referral at least three days prior to death, and another study by Lin et al. [26] found that the median time from PC referral to death was 33 days.

Hospice referral rates are one of ASCO’s QOPI metrics and, in these studies, ranged between 66% and 76% while utilization rates ranged from 38% to 86% [12,30]. The timing of hospice referral and enrollment also varied between studies. Oberndorfer et al. [24] found that while 0% of patients were enrolled in hospice care between six and ten weeks prior to death, hospice enrollment increased to 24% between weeks two and six prior to death and reached 38% in the last two weeks before death. One study by Diamond et al. [23] found that 23% of home hospice GBM patients were enrolled within seven days of death. Kuchinad et al. [30] found that patients were referred to hospice a median of 22 days before death and had a median hospice LOS of 21 days, while Hemminger et al. [29] reported a median time of 18.5 days from hospice enrollment to death and found that nearly 60% of patients had enrolled in hospice within seven days of death.

Lastly, four studies reported hospitalizations at or near the EOL [30,34,35,36]. The percent of patients with a hospitalization in the last month of life ranged from 37% to 42%, with an average LOS of 8.75 days [30,36]. One study reported that among those hospitalized in the last month of life, 34% had an ICU admission [36]. Two studies discussed hospitalizations and found that 20% to 56% of patients spent over 25% of their overall survival time hospitalized, and notably, up to 20% of patients spent 100% of their overall survival time hospitalized [34,35]. Paszat et al. [34] reported that more than half (56%) of GBM patients diagnosed between 1982 and 1994 in Ontario spent over 25% of their overall survival time hospitalized. Among patients spending over 25% of their overall survival time hospitalized, the median cumulative LOS was 31 days, which was almost double compared to 17 days in those spending less than 25% of survival time hospitalized [35].

### 3.4. Location of Death

Six studies included data on location of death (Table 1) [22,28,29,30,32,36]. In three of the studies, death was most common in a home setting, ranging from 39% to 64% of deaths [29,30,32]. In the three other studies, however, death in other healthcare facilities (including hospitals and skilled nursing facilities) was more common, with up to 78% patients dying in such facilities [22,28,36]. Flechl et al. [28] found that of the 45% of patients who desired to die at home, 68% of them died there, while 27% of them died in a hospital, and 5% in hospice. Lastly, across studies, hospice, whether inpatient or at home, was listed as the location of death for 12% to 64% patients [22,28,29,30,32].

### 3.5. Patient-Reported Experiences

Among all studies describing patients’ experiences at the EOL, two described patient-reported outcomes [25,31]. In the study by Seibl-Leven et al. [25], patients completed a self-assessment using the Palliative Outcome Scale to report their PC concerns [37,38]. While “other symptoms” (defined as symptoms other than pain) were of minor importance to this cohort of GBM patients, familial anxiety and patients’ own illness-related anxieties were of high importance. Moreover, when asked to report what problems they have experienced in the recent past, GBM patients most commonly reported experiencing health-related impairments (33%), worries about the future (11%), and loss of everyday skills (10%) [25]. On the other hand, Miranda et al. [31] used the Life Priorities Survey [39] to describe patients’ goals and priorities during EOL. The survey allows patients to designate which goals and priorities are most important to them using Likert scaling and ranking their top five goals and priorities [39]. While “live as long as possible, no matter what” was the priority most frequently ranked in patient’s top three goals (*N* = 9 of 22 patients), three of the 22 said it was “not important at all” [31]. Lastly, all 22 patients reported that “(being) mentally aware” and “(being) independent” were “somewhat to extremely important” [31].

### 3.6. Caregivers’ Experiences

Two studies focused on GBM caregivers’ quality of life, challenges faced, and emotions at the end of the patients’ life [25,28]. Flechl et al. [28] found that 50% of caregivers reported job restrictions that impaired their caregiving abilities, 29% reported that they felt incompletely prepared for their tasks, and 29% reported financial difficulties as a significant barrier. Financial difficulties were associated with caregiver burnout (60%) and reduced quality of life, with impaired quality of life among caregivers being comparable to that reported in GBM patients themselves [28]. In a study by Seibl-Leven et al. [25], financial difficulties (mean Zarit Burden Interview {ZBI} score 0.76 ± standard deviation 1.08) were of lower concern among caregivers, whereas both insufficient time for self (1.36 ± 1.16) and stress between caring and other responsibilities (1.56 ± 1.30) emerged as common challenges. However, the caregivers indicated that they did not feel overly burdened by the patient’s illness-related symptoms (e.g., embarrassment over patient behavior), and they did not endorse a feeling of being “unable to care for relatives any longer” or wishing to leave the “care of relative to someone else” [25].

### 3.7. Study Quality

Using the Newcastle-Ottawa Scale [20,21], five studies were found to be of moderate quality [22,26,34,35,36] and ten were of low quality [12,23,24,25,27,29,30,31,32,33] (Table 1). None were found to be of high quality, because they were not randomized controlled trials. The most common study quality detriment was the “comparability” domain, driven by lack of control groups or matched subjects in study designs. The most common quality strength was in the “outcomes” domain, driven by the use of independent blind assessments or record linking to assess outcomes.

## 4. Discussion

This systematic review suggests low rates of ACP documentation ranging from 4% to 55% for adult GBM patient cohorts in different countries in six separate studies, and that the existing literature on ACP, patient-reported experiences beyond symptomatology, and PC services utilization is sparse, varied, and of low quality. Most studies addressed only disparate aspects regarding EOL needs and experiences among adult GBM patients. Although our literature review did not yield any randomized controlled trials that met the pre-specified eligibility criteria, we did find a published protocol for a forthcoming randomized phase III trial evaluating the effect of early PC for GBM patients [40]. Future additional studies with rigorous design, particularly prospective investigations, are warranted.

Our findings continue to suggest that the adult GBM patient population would benefit from more and better ACP. There are already existing groups pursuing novel programs to better address this deficit, and quality measures used in other cancer populations that could be translated into GBM management. For example, Fritz et al. designed and developed an ACP program for physicians from multiple specialties involved in GBM patient care, as well as for a few GBM patients and their caregiver proxies, to discuss topics including financial concerns and proxy needs [41]. Providers caring for GBM patients may potentially also participate in the ASCO QOPI, which was introduced in 2006 and consists of over 150 quality metrics for oncology practices [42]. While QOPI focuses on clinical practices for melanoma, breast, colorectal, gynecological, lymphoma, lung, and prostate cancers, some of the PC and EOL measures are also applicable to neuro-oncology patients. QOPI EOL metrics include documentation of pain and dyspnea assessments, rates and timing of hospice enrollment and PC referrals as well as the proportion of patients who present to emergency departments or are admitted to intensive care units within the last 30 days of life [19]. According to one study of oncology practices that report and submit data on QOPI EOL measures, each subsequent year of participation led to improved rates of symptom documentation and PC referrals as well as increases in favorable performance related to care of pain compared to peer organizations [43]. Some existing studies of GBM patients already report data related to QOPI EOL measures [12,29,30]. Additional future studies with data on QOPI adherence may be beneficial in characterizing the landscape of ACP and EOL care for adult GBM patients. In the future, QOPI measures may also be established specifically for neuro-oncology practices (e.g., neurological deficits) and lead to improved care for patients with brain cancer at the EOL.

Many studies included in this systematic review focused on healthcare resource utilization, such as length of stay and referrals to PC or hospice. Yet, only a minority of these studies explored the proportion of patients referred and the timing of the referral. In these studies, patients were generally referred to hospice near the EOL, within a range of 3–22 days prior to death. Little data were available from these studies about the decision-making underlying the timing of these referrals and whether these referrals are based on qualitative scales of functional status or disease and treatment course. This systematic review includes studies from multiple countries and healthcare systems that define in different ways the scope of palliative care and hospice services. For example, hospice is regarded as a Medicare benefit in the United States, while other countries include hospice as an extension of available palliative care resources for patients to enroll in. Overall, there is much room for improvement in palliative and hospice services utilization by patients with aggressive and incurable diseases, such as GBM.

Patient and caregiver-reported experiences and concerns are also important considerations of EOL care. Out of all included studies within this systematic review, only a few refer to these outcomes, each utilizing separate methodologies, such as self-assessment surveys like the Palliative Outcome Scale or interview questions. GBM patients most commonly expressed concern with their health, while caregivers had many worries regarding finances according to Flechl et al. [28]. In other studies involving patients with advanced cancer and even serious cases of bacteremia, common patient-reported concerns also centered on psychological distress and limitations on function [44,45,46]. Future studies on GBM patients may further delineate these emotional and physical concerns through use of rigorous qualitative methods to enhance understanding of EOL experiences and needs.

### Limitations

Study limitations include heterogeneity of cohort size and study design, which make reported outcomes difficult to generalize. Selection biases and confounding factors are also inherent to retrospective studies, such as many summarized in this systematic review. In addition, this review is limited by the paucity of studies dedicated solely to adult GBM patients, particularly as many studies do not report outcomes solely for GBM and instead summarize them for patients with all high-grade gliomas as a single cohort.

Furthermore, this systematic review highlights multiple studies that rely on metrics as indicators for quality palliative and goal-concordant care (e.g., rates of ACP documentation and hospice enrollment), which is itself difficult to define and measure [11]. Some have cautioned against focusing on such metrics as the solution to achieving goal-concordant care [47,48], because the metrics may not reflect the quality of palliative care or achieve goal-concordant care. Because measuring outcomes and quality in palliative care still requires standardization via such metrics, this is a limitation inherent to this systematic review.

## 5. Conclusions

Adult GBM patients have a poor prognosis and experience an array of debilitating symptoms due to the incurable and concurrent neuro-degenerative nature of their disease. Proactive advance care planning and appropriate use of palliative care resources are critical aspects of high-quality care for these patients and their caregivers, yet our findings suggest relatively low prevalence of both of these components among GBM patients. The field would benefit from rigorous studies, particularly involving prospective cohorts, to inform future improvements in ACP and EOL care for adult GBM patients as well as to explore other pertinent topics (e.g., whether and which social determinants are associated with utilization of PC, high-quality EOL quality metrics).

## Figures and Tables

**Table 1:** Summary of eligible studies after screening.

Study ID (Country of Study)	Study Design	Study Time Period	Population Description (Sample Size, % Male, Age)	Inclusion/Exclusion Criteria	Quality Assessment	Aims	Pertinent Study Findings
**Paszat et al., 2001** **(Canada)** **[34]**	Population-based retrospective cohort study	1 January 1982–31 December 1994	All patients diagnosed with GBM in Ontario (*n* = 3279, 59.0% male, median age of 61 years).	Include all patients with GBM diagnosed in Ontario between 1/1/82–12/31/1994, as defined by ICD codes on pathology report. Exclude anaplastic astrocytoma or other (non-GBM) histologic type tumors.	Moderate	Report the use of surgery and radiation therapy in GBM in Ontario, survival, and time spent in hospital after diagnosis	Percentage of survival time spent in hospital: <10% of the time: 22.2% 10–24% of the time: 21.4% 25–49% of the time: 17.8% 50–99% of the time: 18.5% 100% of the time: 20%
**Oberndorfer et al., 2008 (Austria)** **[24]**	Retrospective	2003–2008	Inpatients with GBM who died in the Department of Neurology (*n* = 29, 69% male, mean age of 59 years).	Include consecutive inpatients with GBM diagnosis who died in the Department of Neurology at Kaiser Franz Joseph Hospital in Vienna. Exclude patients who were treated for GBM but did not die at the hospital.	Low	Evaluate symptoms, drug treatment, frequency of diagnostic and interventional procedures at EOL	Prevalence of hospice care: 6–10 weeks before death: 0% 2–6 weeks before death: 24% Last 2 weeks before death: 38%
**Lin et al., 2013 (Australia)** **[26]**	Retrospective	1 July 2009–29 February 2012	Inpatients with GBM referred to PC consultation service (*n* =50, 54% male, 86% over 50 years old).	Include inpatients with GBM diagnosis at The Royal Melbourne Hospital who had their first PC involvement during an admission between 7/1/09–2/29/12.	Moderate	Describe PC involvement with GBM population (including symptom burden, allied health involvement)	Median time from GBM diagnosis to PC consult: 111 days Median time from PC referral to death: 33 days Patients with community PC service referral: 40% Patients discharged to inpatient PC unit: 36%
**Flechl et al., 2013 (Austria)** **[28]**	Cross-sectional	2005–2006	Caregivers of adult (age ≥ 18 years) GBM patients and GBM patients (Caregivers: *n* = 52, 33% male, median age 62 years; Patients: *n* = 52, 63% male, median age 63 years).	Include caregivers (defined as defined as next of kin per medical file) of adult (>18 years) GBM patients diagnosed and treated in the Medical University Hospital of Vienna and the Kaiser Franz Josef Hospital in Vienna.	Very Low	Assess caregivers’ perspective on end-of-life phase (last three months) of GBM patients to improve counseling and support	Place of death: Home: 40% Hospice: 12% Other healthcare facility (IP hospital unit, ED, SNF): 48%
**Alturki et al., 2014 (Canada)** **[22]**	Retrospective cohort	2003–2006	Deceased patients or hospitalized patients with primary ICT (*n* = 1065 GBM only, 58.6% male, 86.6% over 50 years old).	Include patients whose death certificate or MedEcho record lists a primary ICT as primary cause of death per histology codes (ICDO-3). Exclude patients who died with—but not because of a—primary ICT.	Moderate	Determine the variability in processes of care in the last 6 months of life experienced by patients dying of primary ICT	Place of death: Home: 8.8% Hospice: 13.2% Other healthcare facility (IP hospital unit, ED, SNF): 77.9%
**Arvold et al., 2014 (United States)** **[35]**	Retrospective cohort	1 January 1999–31 December 2007	Medicare beneficiaries (age 65 years and older) with diagnosis of GBM in SEER registry (*n* = 5029, 51.6% male, 51.6% age 65 to ≤74).	Include Medicare (Part A and B) beneficiaries with GBM diagnosis between 1/1/1999 and 12/31/2009 in SEER regions with coverage through death. Exclude patients without a known diagnosis month, diagnosed at autopsy, or diagnosed without histologic evidence.	Moderate	Understand burden of hospitalization and proportion of time spent in hospital among elderly GBM patients	Discharge disposition to hospice after index hospitalization for GBM: 3.6% Median cumulative days hospitalized from diagnosis to death: 15 days Among patients spending over 25% of remaining life hospitalized: - Proportion of patients: 20.2% - Index hospitalization median LOS: 10 days - Proportion of patients with ≥1 hospitalization after index: 72.3% - Median hospitalizations per patient: 3 - Proportion of patients with ≥1 outpatient visits after index: 93.8% Median cumulative LOS: 31 days
**Pompili et al., 2014 (Italy)** **[32]**	Cross-sectional	January 2012–August 2013	GBM patients receiving at-home assistance at the time of study or who were previously receiving care but died (*n* = 197 total, *n* = 122 GBM only, *n* = 64 GBM patients who died. Among those with GBM who died: 51% male, 45% over age 65).	Include GBM patients who elected to enroll in the home assistance program immediately after first observation or discharge after surgery. Only patients who enrolled between 2012 and first 8 months of 2013 and lived in the Great Highway Ring of Rome were included.	Low	Retrospectively analyze the data from a home assistance program to determine location of death, patient/family satisfaction, cost effectiveness etc.	Prevalence of any ACP documentation: 6% Place of death: - Home: 53.1% - Hospice: 34.4% - Other healthcare facility (IP hospital unit, ED, SNF): 12.5%
**Diamond et al., 2016 (United States)** **[23]**	Retrospective cohort	2009–2013	Adult (age ≥ 18 years) ICT patients admitted to not-for-profit home hospice agency in New York City (*n* = 160 total. GBM only *n* = 104. Among all: 58% male, mean age of 63.4 years).	Include all adult (≥18 years) patients with an ICT (per ICD-9 code) admitted to a home hospice agency in New York City from 2009–2013 who died by the end of 2013, including patients without a histopathological diagnosis but with ICT on imaging. Exclude patients with systemic cancers and brain metastases.	Low	Determine frequency of and characteristics (clinical correlates and sociodemographic features) associated with late hospice referral in primary malignant ICT	Prevalence of late hospice referral: 22%
**Thier et al., 2016 (Austria)** **[33]**	Prospective	2005–2010	Adult (age ≥ 18 years) GBM patients who consecutively died in a hospital neurology ward (*n* = 57, 68% male, mean age at death 59 years).	Include adult (≥18 years) inpatients who died in neurology ward because of GBM between 2005 and 2010 and admission was due to insufficient supportive EOL care in home or other nonspecialized medical departments. Exclude patients whose cause of death was not GBM.	Low	Investigate signs, symptoms, and therapeutic strategies in patients with GBM in the EOL (last 10 days) phase to improve EOL care	Prevalence of any ACP documentation: 4%
**Diamond et al., 2017** **(United States)** **[36]**	Case-control	January 2009–June 2015	Include adult (≥18 years) patients with GBM followed to death at Memorial Sloan Kettering Cancer Center (*n* = 385, 60% male, 53.2% age 60 or above).	Include adult (≥18 years) patients with GBM followed to death at Memorial Sloan Kettering Cancer Center between January 2009 and June 2015 with a histopathologic diagnosis by ICD-9 code and death by the end of study period. Exclude patients only evaluated once, or without a neuro-oncology visit within 60 days of death.	Moderate	Determine factors associated with late hospital admission (defined as admission within one month of death) among patients with GBM	Prevalence of hospitalization within one month of death (“late”): 42.6% Chaplain visit: 59.2% ICU admission (among those with late hospitalization): 34% Place of death in healthcare facility (IP hospital unit, ED, SNF) among those with late hospitalization: 32.0%
**Kuchinad et al., 2017** **(United States)** **[30]**	Retrospective cohort	2009–2014	Randomly selected adult (age ≥ 18 years) GBM patients who received therapy and follow-up care at Johns Hopkins Hospital and/or hospice care at Gilchrist Hospice (*n* = 100, 62% male, median age 64.7 years).	Include adult (≥18 years) GBM patients receiving care at Johns Hopkins Hospital or Gilchrist. Exclude patients with a diagnosis other than GBM, or who did not receive therapy and follow up at Johns Hopkins Hospital.	Low	Retrospectively analyze EOL care for GBM patients at an academic center and compare utilization of these services to national quality of care guidelines, with the goal of identifying opportunities to improve EOL care.	AD documented: 17% Code status documented: 40% Hospice referral: 76% Enrolled in hospice: 86.7% Median LOS in hospice: 21 days Median days before death referred to hospice: 22 days Hospitalization in last 4 weeks of life: 37% Mean LOS per hospitalization: 8.75 days Place of death: - Home hospice: 64.4% - IP hospice: 20.0% - Other healthcare facility (IP hospital unit, ED, SNF): 8.9% - Unknown: 6.7%
**Hemminger et al., 2017 (United States)** **[29]**	Retrospective	1 January 2010–1 May 2015	Patients diagnosed with GBM between 1 January 2010 and 1 May 2015 and who died before 1 November 2015 (*n* = 117, 57.3% male, median age 63 years).	Include patients diagnosed with GBM between 1/1/10 and 5/1/2015 and who died before 11/1/15. Exclude any patient that was alive at the time of analysis or received primary oncologic care at an institution other than the University of Rochester Medical Center.	Low	Evaluate adherence to ASCO’s 5 palliative care quality measures and explore associations with patient outcomes in GBM	ACP documentation by the third oncology visit: 52.1% Health care proxy documentation: 49.2% MOLST form documentation: 36.1% Non-hospital DNR documentation: 1.6% PC consult: 36.8% (Inpatient: 26.5%, Outpatient: 10.3%) Enrolled in hospice >7 days before death: 59.8% Median time from hospice enrollment to death: 18.5 days Place of death: - Home: 38.5% - Home hospice: 17.9% - IP hospice: 22.2% - Other healthcare facility (IP hospital unit, ED, SNF): 13.7% - Unknown: 7.7%
**Miranda et al., 2018 (United States)** **[31]**	Prospective and retrospective chart review	November 2012–February 2015	Control arm participants of the Dana Farber Cancer Institute patient panel participating in the Serious Illness Care Program trial with GBM diagnosis with a “No” on Surprise Question * (*n* = 33, 57% male, median age 55 years).	Include adult (≥18 years) GBM patients in the control arm of the Dana Farber Cancer Institute Serious Illness Care Program trial. Include based on “no” on Surprise Question. Exclude if not English speaking, not receiving primary care at institution, unable to consent and complete periodic surveys, unable to identify family or friend willing to answer surveys.	Low	Describe goals of GBM patients at EOL, describe prevalence timing of SI conversations, and evaluate the quality of SI conversations	Prevalence of an ACP or GoC conversation: 83%
**Seibl-Leven et al., 2018 (Germany)** **[25]**	Prospective explorative field study	January 2014–December 2016	GBM patients treated in- or outpatient and caregivers who completed self-assessment of symptoms and caregiver burden (*n* = 95 GBM patients, 60.2% male, mean age 58.6 years; *n* = 71 caregivers, 31% male, mean age 53.9 years).	Include all GBM patients treated in- or outpatient at the Center for Neuro-Oncology of the University Hospital of Cologne who completed self-assessment of PC symptoms and concerns. Include primary caregivers of GBM patients (defined as living with patient or being in contact ≥2 times per week) who completed self-assessment.	Low	Explore the implementation of self-reported outcome measurements assessing PC symptoms and concerns and caregiver burden in GBM patients	Patients using specialized PC consulting service: 34%
**Pollom et al., 2018 (United States)** **[12]**	Retrospective	2014–2015	Consecutive adult patients (age ≥ 18 years) with newly diagnosed GBM, treated with radiation at Stanford Health Care (*n* = 63, 70% male, median age 63 years).	Include adult (≥18 years) newly diagnosed GBM patients treated with radiation therapy at Stanford Health Care between 2014 and 2015.	Low	Describe ACP documentation and referral to palliative care and hospice among patients with GBM undergoing radiation therapy at am academic center	ACP documentation before death (among decedents): 55% ACP documentation at last follow-up: 54% Living will documented: 29% Durable power of attorney documented: 41% Resuscitation status documented: 46% PC referral: 39% Among those with PC referral, referral within three days of death: 0% Hospice referral: 66% Among those with hospice referral, referral within three days of death: 2%
**Evans et al., 2019 (Canada)** **[27]**	Cross-sectional	2015–2018	Cancer patients participating in INTEGRATE project’ Sunnybrook Health Sciences Centre (GBM only) (*n* = 760 total, *n* = 126 GBM only. Among GBM patients: mean age 62 years).	Include patients receiving care at four cancer centers participating in the INTEGRATE program in Ontario (Sunnybrook Health Sciences Center, Princess Margaret Hospital, The Ottawa Hospital, and the Royal Victoria Regional Health Centre), with GBM, other CNS, lung, head and neck, and gastrointestinal cancers between 2015 and 2018. Included patients were identified with a “no” in response to the Surprise Question *.	Low	Assess implementation of INTEGRATE, a real-world intervention aimed at delivering early palliative care to patients with cancer in Ontario	Prevalence of an ACP or GoC conversation: 83%

* Surprise Question refers to a patient inclusion screening tool where clinicians are asked “Would you be surprised if this patient died within the next year?”; an answer of “no” made the patients eligible for both studies. ACP: advance care planning. AD: advance directive. ASCO QOPI: American Society of Clinical Oncology’s Quality Oncology Practice Initiative. DNR: do not resuscitate. ED: emergency department. EOL: end of life. GBM: glioblastoma. GoC: goals of care. ICD: International Classification of Diseases. ICT: intracranial tumor. ICU: intensive care unit. IP: inpatient. LOS: length of stay. MOLST form: medical orders for life-sustaining treatment form. PC: palliative care. SEER: Surveillance, Epidemiology, and End Results database. SI: serious illness. SNF: skilled nursing facility.

## Data Availability

No new data were created or analyzed in this study. Data sharing is not applicable to this article.

## Appendix A. Search Strategies

Scopus TITLE-ABS-KEY ((Glioblastoma* OR ((astrocytoma OR astrocytomas OR astrocytic OR glioma) pre/4 “grade IV”) OR “GBM” OR spongioblastoma) AND (“terminal care” OR “end of life” OR “end of life care” OR “EOL” OR “End-of-life” OR “advance care planning” OR “advance directive*” OR “advanced care” OR “advance care” OR “advance health care planning” OR “advance medical planning” OR “advanced care planning” OR “attitude to death” OR “withholding treatment”)).

Cochrane Library (glioblastoma OR glioma OR astrocyte* OR spongioblatoma OR “GBM”) AND (“terminal care” OR “end of life” OR “end of life care” OR “EOL” OR “End-of-life” OR “advance care planning” OR “advance directive*” OR “advanced care” OR “advance care” OR “advance health care planning” OR “advance medical planning” OR “advanced care planning” OR “attitude to death” OR “withholding treatment”).

Embase (‘glioblastoma’/exp OR glioblastomas OR glioblastoma OR ((astrocytoma OR astrocytomas OR astrocytic OR glioma) NEXT/4 ‘grade iv’) OR ‘gbm’:ti,ab,kw OR spongioblastoma) AND (‘terminal care’:ti,ab,kw OR ‘terminal care’/exp OR ‘end of life’:ti,ab,kw OR ‘end of life care’:ti,ab,kw OR ‘eol’:ti,ab OR ‘end-of-life’:ti,ab,kw OR ‘advance care planning’/exp OR ‘advance directive*’:ti,ab,kw OR ‘advance health care planning’:ti,ab,kw OR ‘advance medical planning’:ti,ab,kw OR ‘advanced care planning’:ti,ab,kw OR ‘attitude to death’ OR ‘treatment withdrawal’) NOT (‘glomerulus basement membrane’/exp OR ‘glomerulus basement membrane’).

Cochrane Library (glioblastoma OR glioma OR astrocyte* OR spongioblastoma OR “GBM”) AND (“terminal care” OR “end of life” OR “end of life care” OR “EOL” OR “End-of-life” OR “advance care planning” OR “advance directive*” OR “advanced care” OR “advance care” OR “advance health care planning” OR “advance medical planning” OR “advanced care planning” OR “attitude to death” OR “withholding treatment”).
